# *DMD* antisense oligonucleotide mediated exon skipping efficiency correlates with flanking intron retention time and target position within the exon

**DOI:** 10.1080/15476286.2023.2254041

**Published:** 2023-09-04

**Authors:** Remko Goossens, Nisha Verwey, Yavuz Ariyurek, Fred Schnell, Annemieke Aartsma-Rus

**Affiliations:** a Department of Human Genetics, Leiden University Medical Center, Leiden, The Netherlands; b Leiden Genome Technology Center, Leiden University Medical Center, Leiden, The Netherlands; c Sarepta Therapeutics, Inc, Cambridge, MA, USA

**Keywords:** Duchenne Muscular Dystrophy, Dystrophin, DMD, Antisense oligonucleotides, Exon skipping, AON, ASO

## Abstract

Mutations in the *DMD* gene are causative for Duchenne muscular dystrophy (DMD). Antisense oligonucleotide (AON) mediated exon skipping to restore disrupted dystrophin reading frame is a therapeutic approach that allows production of a shorter but functional protein. As DMD causing mutations can affect most of the 79 exons encoding dystrophin, a wide variety of AONs are needed to treat the patient population. Design of AONs is largely guided by trial-and-error, and it is yet unclear what defines the skippability of an exon. Here, we use a library of phosphorodiamidate morpholino oligomer (PMOs) AONs of similar physical properties to test the skippability of a large number of *DMD* exons. The *DMD* transcript is non-sequentially spliced, meaning that certain introns are retained longer in the transcript than downstream introns. We tested whether the relative intron retention time has a significant effect on AON efficiency, and found that targeting an out-of-frame exon flanked at its 5’-end by an intron that is retained in the transcript longer (‘slow’ intron) leads to overall higher exon skipping efficiency than when the 5’-end flanking intron is ‘fast’. Regardless of splicing speed of flanking introns, we find that positioning an AON closer to the 5’-end of the target exon leads to higher exon skipping efficiency opposed to targeting an exons 3’-end. The data enclosed herein can be of use to guide future target selection and preferential AON binding sites for both DMD and other disease amenable by exon skipping therapies.

## Introduction

Most gene transcripts undergo pre-mRNA splicing. In this process, the coding sequences of gene transcripts (exons) are joined together, while the non-coding intronic sequences are removed by the spliceosome in the form of lariat RNA. Introns are generally longer than exons and intron size ranges anywhere from less than 100 base pairs to over 100 kb. A notable example is the *DMD* gene, which encodes the dystrophin protein. The full-length transcripts are 2.21 Mb long, and introns make up 99.3% of the transcript size, varying in size between 107 bp and over 248 kb.

Mutations in the *DMD* gene that abolish production of functional dystrophin protein cause Duchenne muscular dystrophy (DMD), which manifests as progressive muscle wasting and loss of muscle function starting in young boys and leads to premature death, generally in the 3^rd^ or 4^th^ decade of life [1]. The majority of mutations causing DMD are deletions of one or multiple exons, leading to abolished expression or dysfunctional dystrophin protein. A hotspot of reading frame disrupting *DMD* deletions is located between exon 43 and 55 [2,3]. Mutations in *DMD* can also cause Becker muscular dystrophy (BMD), which is characterized by a milder phenotype than DMD, but BMD mutations generally maintain the reading frame of *DMD* [2]. For about 55% of *DMD* mutations in DMD patients, it would be feasible to restore the reading frame by skipping a neighbouring exon using antisense oligonucleotides (AONs) [4]. Currently, there are 4 FDA approved AON therapies for DMD, all of which target an exon in the exon 43–55 hotspot region [5]. The purpose of these AONs is to induce exon skipping of exons 45, 51 or 53, which when applied to patients with a compatible mutation in *DMD*, can restore the reading from to generate a shorter, partially functional dystrophin protein as seen in BMD. However, with the currently approved AONs only about 30% of DMD patients can potentially be treated, meaning that a majority of DMD patients do not yet benefit from the current therapies. The development of different AONs necessary to skip the entire range of skippable *DMD* mutations is however a time consumable and difficult endeavour.

While AON design is guided by practical knowledge available in the scientific community [6], it is still unclear what truly designates a good targeting site for an AON to induce exon skipping. While studies have been published which attempt to catalogue the skippability of various *DMD* exons [7], these results cannot be easily extrapolated as experimental design of the AONs, as well as coverage per exon, varies significantly. Furthermore, this data also showed that some exons are more difficult to skip than others, with the most difficult exons requiring combinations of AONs [7]. Based on retrospective analysis of effective and ineffective AONs, it is known that e.g. increased AON length, GC content and melt temperature play a role in AON efficacy [7–10]. Therefore, it is difficult to compare which other parameters, such as targeting location, would make an AON reliably skip an exon.

Next to clinical necessity, using *DMD* as a model gene to study AON targeting is convenient as ample data is available about dystrophin transcript processing. These data indicate that *DMD* is non-sequentially spliced [11], meaning that some introns are removed by the spliceosome earlier than an upstream intron which was transcribed before it by RNA polymerase II (Pol II). Based on our previously generated data, we classified that some of these introns are ‘slow’, while other introns are ‘fast’, concerning their splicing dynamics. This spatiotemporal model allows us to select sets of *DMD* exons flanked at either their 5’- and 3’-side by these ‘slow’ or ‘fast’ introns, and assess whether this characteristic influences the skippability of the exon. This model also allows us to assess whether targeting the AON itself more proximal or distal within the target exon is beneficial for exon skipping strategies, and whether this targeting location is dependent on the intron-class that flanks it on the target site.

We present here the first *in vitro* study where a large set of phosphorodiamidate morpholino oligomers (PMOs) with similar physical characteristics, such as melting temperature, GC content and free energy to form secondary structures, are used to determine optimal targeting of an exon to induce exon skipping. Our data indicates that exon skipping strategies have a significantly higher chance of successfully skipping an out-of-frame exon when the preceding 5’-intron is retained in the transcript for a longer amount of time. The speed at which the downstream 3’-intron is spliced does not seem to influence skipping efficiency. Furthermore, in the vast majority of targeted exons, there is a significantly higher efficiency of exon skipping when the AON is designed to target the proximal (5’) region of the exon. The data and AON sequences contained herein can be used to guide exonic target selection for Duchenne and our observation adds another design feature that can be taken into consideration when targeting various exons in diseases which are amenable for AON mediated therapies.

## Results

### Exon selection and PMO design

Non-sequential splicing of the *DMD* gene [11], as well as the different splicing speed of its introns could have consequences for the efficiency of AON-mediated exon skipping strategies with a potential correlation between splicing dynamics and skippability of the exon. Various exons of *DMD* are flanked on their upstream 5’-end splice site by an intron, which is retained in the transcript relatively longer (slow intron) or shorter (fast intron). From here on, these intron classes will be referred to as 5’-Slow and 5’-Fast. Similarly, we denote the nature of the downstream 3’-end intron as 3’-Slow and 3’-Fast to indicate that these introns were found to be retained relatively longer or shorter, respectively.

**Figure 1.**
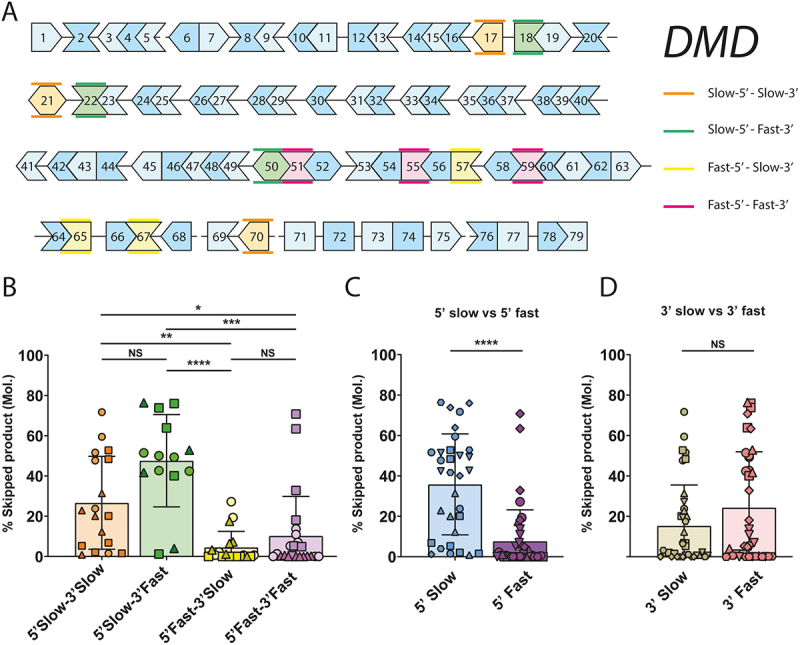
*DMD* exons flanked by a slow upstream intron are more susceptible to exon skipping A: Graphical representation of the splicing order of introns in the *DMD* gene (adapted from [11]). Introns denoted by a line between exons are spliced slow, while exons shown directly adjacent are separated by ‘fast introns’. Different exon classes based on flanking introns are shown in orange, green, yellow and pink as indicated on the right. Different shades of blue are a visual aid with no relevance to the hypothesis. Continuity of the protein coding sequence reading frame is indicated by the matching shapes of the exons. B: Exon skipping efficiency of the *DMD* transcript with AONs for various exons as indicated in A. Each shape represents the average of two independent nucleofected samples, while each unique shape per bar corresponds to AONs targeting the same exon. Skipping efficiency was determined as the molar ratio of skipped product over total (full-length+skipped) product in an RT-PCR analysis using suitable primers for each exon. The exons belonging to the 5’Slow-3’Slow and 5’Slow-3’Fast classes show significantly higher exons skipping efficiency compared to 5’Fast-3’Slow and 5’Fast-3’Fast. C: Data presented in B was reanalysed and grouped to separate exons based on their upstream intron class as indicated. 5’Slow exons show significantly higher skipping efficiency compared to 5’Fast. D: Data presented in B was reanalysed and grouped to separate exons based on their downstream intron class as indicated. 3’Slow exons and 3’Fast exons show no difference in skipping efficiency. **Error bars**: SEM. (*: *P* value < 0.05, **: *P* value < 0.01, ***: *P* value < 0.001, ****: *P* value < 0.0001, NS: Not-significant – B: One-Way ANOVA, C/D: Mann-Whitney).

For each of the potential combination of flanking intron classes (5’Slow +3’Slow, 5’Slow +3’Fast, 5’Fast +3’Slow and 5’Fast +3’Fast), we selected 3 out-of-frame exons of the *DMD* gene for which we designed PMO AONs (Figure 1A). To prevent efficiency bias from the physical properties of the AON, we designed panels of PMOs with similar physical properties, such as nucleotide count, GC content, melting temperature and potential free energy for forming secondary structures. We considered every possible 24/25-mer AON for eligible exons, resulting in 11.213 potential AONs. After calculating average GC (43.98% ±9.70%) content and melting temperatures (66.76°C ± 6.84°C), we only took PMOs which were within 1 SD of the average GC content and melting temperature into consideration. As a basic design constraint: PMOs with a GC content of <40% or >60% or any stretch of three or more subsequent G/C nucleotides were not considered. After exclusion of AONs with unfavourable predicted free energy and secondary structure we had a final selection of eligible PMOs with an average GC content of 47.38% ±2.73% and melting temperature of 69.06°C ± 2.01°C. From this list we selected sets of PMOs for the various exons corresponding to the flanking intron classes described above. Namely: exons 17, 21 and 70 (5’Slow +3’Slow), exons 18, 22 and 50 (5’Slow +3’Fast), exons 57, 65 and 67 (5’Fast +3’Slow) and exons 51, 55 and 59 (5’Fast +3’Fast) (Figure 1A, Supplementary table S1). Larger exons are covered by a larger amount of PMOs (~1 PMO/30 nucleotides) to prevent a bias of coverage based on exon length. Overlap between PMO target sequences was prevented as much as possible. Human splicing finder (HSF) was used to determine whether an overt bias for coverage of exonic splicing enhancer (ESE) motifs occurred for certain designed AONs [12], but it was determined that most dystrophin exons are densely covered in predicted ESEs and that AONs covered a roughly equal amount of sites (data not shown).

### Optimization of PMO delivery to myotube cultures

To facilitate reliable and reproduceable delivery of the PMOs to the control myoblast line we used electroporation in an Amaxa 4D nucleofector X unit with 16-well nucleocuvette strips [13]. We validated the delivery of PMOs using this system in our cell line with a single exon 51 targeting PMO, using various buffer systems and pulse programs. We selected the condition with the highest detectable skipping of exon 51 using RT-qPCR (Supp. Figure S1A), and used this condition (Buffer set P1, pulse program CM-137) for the remainder of the study. Electroporation of myoblasts still allows for differentiation to *DMD* expressing myotubes, as determined by RT-qPCR for myogenic markers *MYOG* and *MYH3* (Supp. Figure S1A). Expression of *MYOG* was reduced for all conditions using the P1 buffer system compared to unpulsed controls, although cell survival seemed largely unaffected. We also compared the efficiency of nucleofection with other methods of antisense oligonucleotide delivery such as gymnosis [14], calcium enrichment of medium [15] and endo-porter reagent [16] (Supp Figures S1B, S1C). However, in our hands nucleofection was the most efficient method for high throughput delivery with the smallest amount of PMO required (ie. 1 µl of a 1 mM PMO stock per sample). The foremost used method for semi-quantification of exon skipping PCR products is the Agilent Bioanalyzer 2100 using a DNA-1000 chip. To facilitate the high amount of generated samples, we compared the Bioanalyzer 2100 data with data generated by the Agilent Femto pulse system, which allows higher automated throughput of samples. Running the same RT-PCR sample on both systems showed no discernable differences in estimated exon skipping efficiency (Supp. Figure S1D).

### Exons with slow 5’-introns appear to be more skippable

We electroporated the set of PMOs targeting the 12 selected exons outlined above in HC myoblasts and allowed cells to differentiate to myotubes. After RT-PCR and analysis of exon skipping efficiency (Supp. Figure S2), we observed that there was a clear, statistically significant, difference in skipping efficiency between the different exon classes (Figure 1B). The 5’Slow-3’Slow and 5’Slow-3’Fast showed higher average skipping efficiencies than the 5’Fast-3’Slow and 5’Fast-3’Fast exon classes. No significant difference was observed between 5’Slow-3’Slow and 5’Slow-3’Fast, or between 5’Fast-3’Slow and 5’Fast-3’Fast. Finally we reanalyzed this data and grouped the exons solely based on splicing speed of their 5’- or 3’-flanking intron. Here, we only observed a significant difference in skipping efficiency when grouping for the splicing speed of the 5’-flanking intron (Figure 1C), but not the 3’-flanking intron (Figure 1D). Together, this data shows that a longer retention of the upstream intron has a positive effect on AON-mediated exon skipping efficiency.

### An AON targeting the 5’-end region within the exon is more efficient

**Figure 2.**
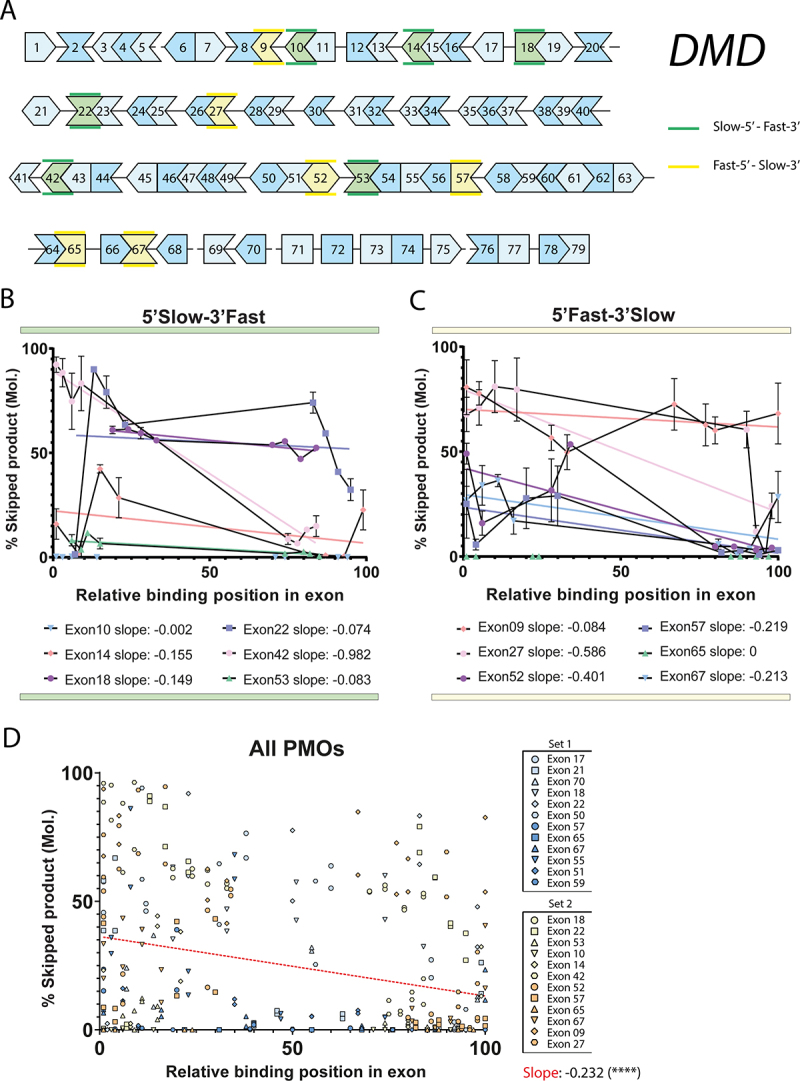
Targeting the 5’-end of an exon leads to higher exon skipping efficiency A: Graphical representation of the splicing order of introns in the *DMD* gene (adapted from Gazzoli et. al. 2016). Introns denoted by a line between exons are spliced slow, while exons shown directly adjacent are separated by ‘fast introns’. Different exon classes targeted for AON mediated exon skipping based on flanking introns are shown in green and yellow as indicated on the right. Different shades of blue are a visual aid with no relevance to the hypothesis. Continuity of the protein coding sequence reading frame is indicated by the matching shapes of the exons. B: Exon skipping efficiency of the *DMD* transcript with AONs for various exons from the 5’Slow-3’Fast flanking intron class as indicated in A. Skipping efficiency was determined as the molar ratio of skipped product over total (full-length+skipped) product in an RT-PCR analysis using suitable primers for each exon. Individual points indicate the average of two independently nucleofected samples. The X-axis represents the possible window of targeting a 25-mer AON within the exonic sequence of the indicated exon, scaled from 0 to 100 for each exon to normalize exon size. Lines matching the symbol colours represent the results of a linear regression analysis of the skipping efficiency as a function of the targeting position. Individual slopes of each regression analysis are indicated below the plot. C: Exon skipping efficiency of the *DMD* transcript with AONs for various exons from the 5’Fast-3’Slow flanking intron class as indicated in A. Plots, axis and analysis description are identical to the plot presented in B. D: Reanalysis and summarizing plot of the *DMD* exon skipping efficiencies of all AONs used in figures 1B, 2B and 2C, as a function of their position within the exon as indicated on the X-axis. Datasets and exons from figure 1 and figure 2 are indicated in the legend on the right. Linear regression analysis (red line) shows a negative slope, indicating that in general, an AON targeting closer to the 5’-end of the exon will be more efficient at skipping the target exon than a more distally targeted AON. **error bars**: SEM. (****: *P* value < 0.0001 – Linear regression).

To test whether the optimal target within an exon was determined by the presence (5’ or 3’) of slow introns, we designed another set of PMOs, now only using 5’Slow-3’Fast (exon 10, 14, 18, 22, 42 and 53) and 5’Fast-3’Slow (exon 9, 27, 52, 57, 65 and 67) exon classes (Figure 2A, Supp table S1). We aimed for 8 AONs per exon, positioning 4 AONs in the proximal 30% and 4 AONs in the distal 30% of the exon. Using these requirements, it was not feasible to select only out-of-frame exons while still adhering to our design criteria for PMO similarity. Therefore, we selected 6 *DMD* exons for each class from both out-of-frame (exon 18, 22, 52, 53, 57, 65 and 67) and in-frame exons (exon 9, 10, 14, 27 and 42).

After delivery of this set of PMOs to HC myoblasts and differentiation to *DMD* expressing myotubes, we analysed skipping efficiency of each PMO as before, using suitable RT-PCR primer sets for each targeted exon. The relative binding position of each of the AONs was calculated as the most proximal or distal exonic position possible for a 25-mer AON on an arbitrary 1–100 scale, and skipping efficiency was plotted on this coordinate for the corresponding PMO (ie. the starting base of a PMO binding site was divided by the length of the exon, subtracted by the PMO length (24/25)). For exon 10 (exon length 189 bp) this means that a 25-mer PMO on either position 1 of the scale (H10A(+01 + 25)) or position 100 of the scale (H10D(25 + 01)), respectively, bind nucleotides 1 through 25 or 165 through 189 of this exon. When analysing the skipping efficiency of PMOs in the 5’Slow-3’Fast category (Figure 2B) or 5’Fast-3’Slow category (Figure 2C), we observed a trend of higher skipping efficiency closer to the 5’-end of the exon for almost all exons tested. This was confirmed by regression analysis of the skipping efficiency of each exon, which showed a negative slope for all skippable exons (Figure 2B, C and Supp. Figure S3). Exons not abiding this observation were exon 65 and 10, which showed no skipping for any of the individual PMOs tested (Supp. Figure S3 and Supp. Figure S4).

This correlation between AON targeting site and skipping efficiency was also observed when we combined the data of all PMOs used in this study (Figures 1 and 2) and plotted the regression analysis (Figure 2D). We noted a significant negative trend towards the 3’ of the exon, indicating that in general, an AON targeting the 5’-end region of an exon has higher potential of being effective, regardless of the flanking introns splicing dynamics.

Out-of-frame transcripts might be unstable, causing them to be degraded by non-sense mediated decay (NMD), a cellular mechanism used to degrade mRNA molecules which contain premature stop-codons. Specifically, degradation of the *DMD* transcript has been described to occur in both healthy control cells as well as DMD patient derived cells [17,18]. Degradation by NMD could cause a bias when targeting both in-frame and out-of-frame exons. We plotted the skipping efficiency for AONs targeting in-frame exons compared to out-of-frame exons, but no significant difference was observed (Supp. Figure S3C). Furthermore, in-frame skipping exons do not seem to fully abide by the earlier observation that a slow-fast exon is skipped more efficiently (Supp. Figure S3D&E), while the data is reinforced for out-of-frame skipping exons (Supp. Figure S3D&F) which show a highly significant correlation with their upstream intron splicing speed. It should be pointed out, however, that the sample size of out-of-frame exons is larger in our analysis (5 in-frame against 7 out-of-frame), which can skew statistical analysis.

Finally, it is known that there are various types of exonic splicing enhancers (ESEs), which can influence the ability of the spliceosome to correctly identify and excise introns [12]. However, as all dystrophin exons are densely populated with predicted ESEs, we did not see a clear correlation between a PMOs coverage of ESEs and its ability to efficiently induce exon skipping. This is exemplified by a comparison of a set of PMOs which induce relatively high exon skipping levels (exon 18) compared to a set of poor exon skipping inducing PMOs (exon 59) (Supp. Figure S5), where the less performing PMOs still overlap with multiple ESE motifs.

### Exon skipping can lead to transcript loss in control cells

**Figure 3.**
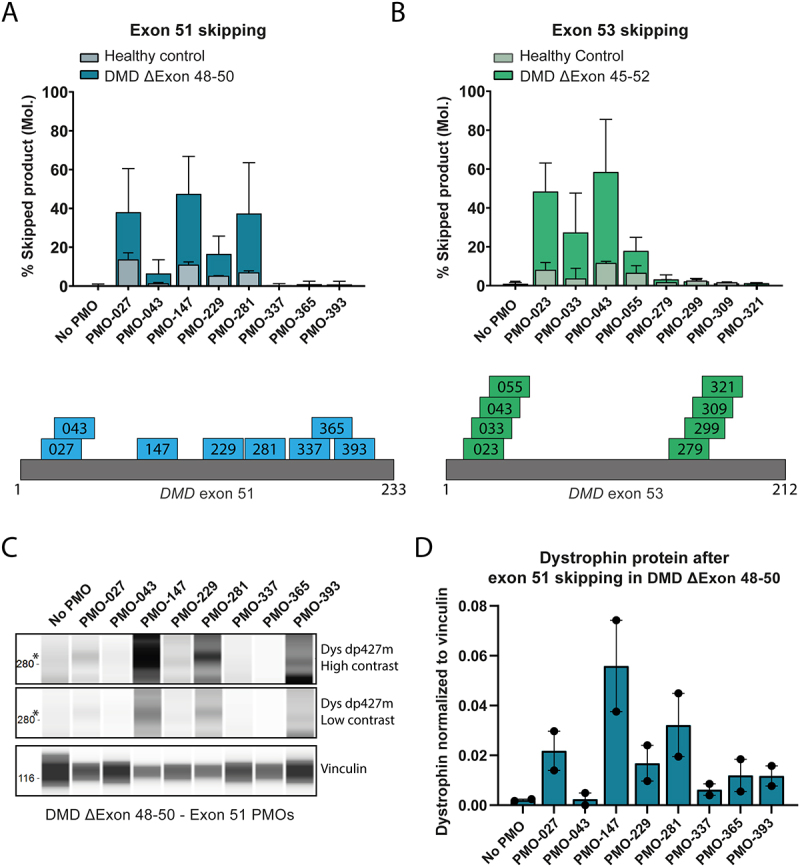
Disruption or restoration of the reading frame due to exon skipping does not change relative compared efficiency A: Exon skipping efficiency of exon 51 of the *DMD* gene in HC myotubes (light blue, front bars) and in DMD patient (ΔExon 48–50) myotubes (dark blue, back bars) after treatment with exon 51 targeting AONs as indicated. Skipping of exon 51 in healthy cells will lead to an out of frame transcript, while in the patient cells skipping of exon 51 will lead to an in-frame transcript. Skipping efficiency was determined as the molar ratio of skipped product over total (full-length+skipped) product in an RT-PCR analysis using suitable primers for each exon. Binding position of the indicated PMOs on the exon 51 sequence (grey bar) is shown below (to scale). B: Exon skipping efficiency of exon 53 of the *DMD* gene in HC myotubes (light green, front bars) and in DMD patient (ΔExon 45–52) myotubes (dark green, back bars) after treatment with exon 53 targeting AONs as indicated. Skipping of exon 53 in healthy cells will lead to an out of frame transcript, while in the patient cells kipping of exon 53 will lead to an in-frame transcript. Skipping efficiency was determined as the molar ratio of skipped product over total (full-length+skipped) product in an RT-PCR analysis using suitable primers for each exon. Binding position of the indicated PMOs on the exon 53 sequence (grey bar) is shown below (to scale). C: Digital western blot image of dystrophin (dys) dp427m (asterisk) signal captured by Wes capillary immunoassay after treatment of DMD patient (ΔExon 48–50) myotubes with exon 51 targeting PMOs as indicated. A high and low contrast image of dp427m are shown. Vinculin was probed in duplex as a housekeeping protein for normalization purposes. D: Quantification of Wes data shown in C. Dystrophin signal was normalized to Vinculin. Y-axis consists of arbitrary units, *N* = 2.

We then aimed to investigate whether the low levels of exon skipping observed in control myotubes for exon 51 and 53 could be partially explained by the fact that these experiments would generate an out-of-frame *DMD* transcript, which could be a target for NMD. We electroporated sets of exon 51 or exon 53 targeting PMOs in two DMD patient derived cell lines. These cells harbour a deletion of *DMD* exon 48–50 (line 8036) [19] or exon 45–52 (line 6311) [20], making them amenable for reading-frame restoration by skipping of exon 51 or exon 53, respectively. When comparing the exon skipping levels in controls observed for exon 51 (Figure 3A, lighter bars) or exon 53 (Figure 3B, lighter bars) with the respective DMD cell lines (Figure 3A,B darker bars), it is apparent that the majority of targeting AONs result in higher exon skipping levels in DMD patient derived cell lines. This indicates that the detectable levels of exon skipping in control cells might be underestimated due to degradation of the skipped transcript. However, trends in AON efficiency remain the same between control and patient cells indicating that the relative skipping efficiency can still be deduced by skipping out-of-frame exons in control cells. This is also suggested by comparing levels of *DMD* transcript as measured by RT-qPCR, using primers up- and downstream of the targeted exons (Supp. Figure S6). As an example, we observe that in control cells, the more efficient AONs skipping exon 51 (Figure 3A), such as PMO-027, PMO-147 and PMO-281 show reduced transcript levels when measuring Exon55–56 (Supp. Figure S6A) in these samples. Indicating that there is a correlation between AON efficacy and loss of transcript. This effect was not observed for exon 53 (Supp. Figure S6B).

### Dystrophin protein recovery by exon 51 PMOs in DMD cells correlates to exon skipping efficiency

The ultimate goal of exon skipping in *DMD* is to generate a shorter, but functional dystrophin protein in patients muscle cells. As a proof of principal, we tested whether the exon skipping efficiency we observed on mRNA level for our set of exon 51 skipping PMOs, also translated to the recovery of dystrophin protein in the DMD exon Δ48–50 patient cell line. Using a capillary western immunoassay system (Wes), we visualized dystrophin protein recovery in myotubes after electroporation of exon 51 targeting PMOs (Figure 3C). Quantification of the normalized dystrophin protein signal (Figure 3D) shows a pattern of protein recovery similar to the exon skipping efficiency of the same PMOs on mRNA level (Figure 3A). A serial dilution of healthy control cell lysates was used to generate a standard curve and assess the detection limit and specificity of the Wes system (Supp. Figures S7A &amp; S7B). The dystrophin signal visualized by Wes shows a lower molecular weight than the expected 427 kDa for full length dystrophin (Figure 3C, Supp. Figure S7A), as has been reported previously [21].

### Nonsense mediated decay may affect detectable skipping efficiency

We noticed that skipping of exon 51 and exon 53 was more efficient in DMD patient cells, where exon skipping restores the reading frame, than in HC cells, where exon skipping disrupts the reading frame. To test whether NMD might play a role in this observation, both HC and DMD myocytes were nucleofected with PMOs, after which cells were treated with cycloheximide (CHX), an inhibitor of NMD. As a proof of principle, we also included samples nucleofected with an equimolar mix of all 12 available exon 65 targeting PMOs, at the same end concentration (Supp. Figure S8A). We aimed to see whether a combination of multiple PMOs that are individually ineffective at inducing exon skipping could still work when combined for a more skipping-resistant exon such as exon 65. The mix of a dozen PMOs leads to minor levels of skipped exon 65, while none of the individual PMOs resulted in detectable levels of exon skipping. Treatment of exon 65 targeting PMO nucleofected HC cells with CHX causes an increase in detected exon 65 skipped *DMD* transcript, indicating that NMD might degrade the skipped product, leading to underestimation of skipping efficiency of out-of-frame exons. However, this effect was less pronounced in the DMD exon Δ45–52 cells, and completely absent in the DMD exon Δ48–50 line (Supp. Figure S8A). RT-qPCR of the *DMD* gene in these samples show a similar, stabilizing effect on the *DMD* transcript, with a modest increase in the total detected expression level (Supp. Figure S8B). Similarly, exon skipping of exon 51 and 53 in the HC (Supp. Figure S8C and S8D) and the two DMD lines (Supp. Figure S8E and S8F) and treatment with CHX yielded inconclusive results. While HC cells showed a slight increase of detectable skipped *DMD* after CHX, DMD cells showed lower detectable skipping efficiency for some PMOs. This result is not dissimilar to previous reports claiming that blocking NMD in DMD cells does not stabilize the *DMD* transcript [18].

## Discussion

In this study we aimed to elucidate the connection between the efficiency of AON mediated exon skipping, and the splicing characteristics of the introns neighbouring the target exon. Our study focused on the *DMD* gene transcripts in human muscle cells, as there is great potential for therapeutic use of AONs in treating DMD. For this purpose we used a library of 153 custom designed and individually tested *DMD* targeting PMOs with similar physical properties. We found that targeting an out-of-frame exon of which the upstream (5’-end) intron is retained in the transcript longer (a ‘slow intron’) leads to generally higher efficiency of exon skipping than when the exon is preceded by a rapidly spliced (‘fast’) intron. There seems to be no correlation between the splicing speed (‘slow’ nor ‘fast’) of the downstream (3’-end) intron on an exons skippability. Furthermore, we analysed whether targeting an AON closer to a 5’- or 3’-end of either a ‘fast-slow’ or ‘slow-fast’ exon would lead to a difference in exon skipping efficiency. We observed a clear indication that targeting a sequence closer to the 5’-end of the exon leads to more efficient exon skipping than targeting its 3’-end. Our study is the first to systematically assess optimal exons and optimal target sites within exons for AON-mediated exon skipping. Other (retrospective) studies [7–10] on exon skipping efficiency suffered from vastly different physical properties and/or chemistry of the AONs, making comparisons of targeting site efficiency hard to interpret. Our study specifically aimed at reducing differences that could be caused by physical differences in the AONs, by designing PMO AONs of highly similar characteristics such as length, melting temperature, GC content and free energy potential.

A discrepancy was observed in the efficiency of skipping ‘slow-fast’ exons, depending on whether the resulting transcript is in-frame or out-of-frame. Here, out-of-frame exons abide by the observation that skipping efficiency is correlated with a slow 5’-flanking intron while in-frame exons do not. It would be of interest for future studies to assess, whether this observation is circumstantial or correlated with induced frameshifts in the transcript upon splicing, especially as most therapeutic exon skipping of interest for Duchenne concerns out-of-frame exons.

Currently, four different *DMD* targeting AONs have been FDA approved for the treatment of Duchenne: Eteplirsen (exon 51), Golodirsen (exon 53), Viltolarsen (exon 53) and Casimersen (exon 45) [5]. Eteplirsen (30-mer) targets nucleotides 66 through 95 of the 233 bp exon 51. Golodirsen (25-mer) and Viltolarsen (21-mer) respectively target nucleotides 36 through 60 or 56 of the 212 bp exon 53. Casimersen’s (22-mer) targeting sequence extends from the last 3 nucleotides of intron 44 through the 19^th^ nucleotide of the 176 bp exon 45. Hence, the targeting sites of these therapeutic AONs largely are in accordance with our findings that targeting the 5’-end of an exon would be a preferential targeting site over a 3’-end targeting AON. However, our AON design never included intronic sequences, so the possibility remains that AONs spanning the intron-exon junction are more efficient than pure exonic targeting AONs.

Our initial screening method only sought to include out-of-frame exons to test our hypothesis on the influence of intronic retention times. While this approach leads to more potential to use the dataset in the development of AONs to restore the *DMD* reading frame in currently untreatable cases, it does lead to the generation of out-of-frame transcripts in the healthy control cell line used. It is therefore likely that nonsense mediated decay could degrade skipped transcripts, leading to an underestimation of exon skipping potential of an NMD-triggering AON. Indeed, we observe that inhibition of NMD by a six hour CHX treatment in HC cells treated with certain exon 51 or 53 targeting PMOs increases the observed amount of exon skipping as determined by RT-PCR. Levels of *DMD* transcript are also increased in HC-cells treated with CHX as seen by RT-qPCR, confirming previous work suggesting that *DMD* is also degraded through NMD in a wildtype situation [17,18]. Intriguingly, the *DMD* stabilizing effect was not observed in muscle cells from DMD patients that would be amenable to exon skipping therapies with exon 51 or 53 targeting PMOs. In these cells, while skipping levels were markedly higher than in HC cells, no additional benefit was seen from a combination of CHX and AONs, suggesting that the *DMD* transcript in DMD patients is not stabilized in our treatment window of six hours. Previously published data on inhibition of NMD by CHX treatment in DMD patient cells used a treatment time of 24 hours [18]. This suggests that potentially not enough out-of-frame, and therefore NMD-substrate, dystrophin transcripts have accumulated in our samples yet, as this transcript can take as long as 16 hours to be transcribed.

The exons included in this study were positioned over the entirety of the *DMD* gene as far as our described design constraints allowed. Transcription of *DMD* is suggested to take as long as 16 hours, and there is a reported imbalance between the abundance of the 5’- and 3’-end of the transcript [22]. The 3’-end of *DMD* being underrepresented indicates that potentially, transcription is properly initiated but unfinished. As splicing of *DMD* occurs co-transcriptionally, there might be a correlation between the transcription of the RNA molecule and the possibility to skip an exon, as a proximal exon would be more abundantly present in cells. However, we did not see a correlation between exon skipping efficiency of our tested PMOs and the location of the exon in the gene. e.g.: the skippability of exon 17 and 70 is relatively high, while the skippability of exon 10 and 65 was low.

The splicing maps indicating the splicing order of introns of the *DMD* gene were based on results we previously generated in HC cells [11]. It is yet unclear what properties make an intron ‘slow’ or ‘fast’. If this information is contained within the intron itself, it is possible that a large deletion encompassing part of an intronic region will alter the splicing characteristic of the remaining flanking exons. That is, an exon originally flanked by a slow intron might be flanked by a fast intron upon genomic rearrangement in a DMD patient. Whether this occurs would most likely highly depend on the exact breakpoint of the mutation, which is often unique in each patient [2,4]. Whether this ‘intronic-splice-speed-switching’ actually occurs in DMD and whether this would have consequences for AON mediated therapeutics remains unknown until the nature of ‘slow and ‘fast’ introns are elucidated further. DMD patient cells with the same deleted exon, but carrying unique intronic rearrangements, could be treated with an AON library as we present here to determine exon skipping efficiency. Overlaying the intronic deletion pattern with exon skippability might then lead to the discovery of new motifs in the intron, which could be highly valuable information in clinical and pre-clinical research on Duchenne therapies, and fundamental understanding of the splicing process.

In conclusion, our data and tested AON sequences can provide new insights into preferred selection of exon skipping target exons. The observations of splicing speed-related exon skipping efficiency can be taken into consideration when selecting the next exon skipping therapy candidate in DMD and other applicable diseases. Moreover, we show in an unbiased manner that generally, the optimal exon targeting region is the 5’-end, which can guide future design for AON based exon skipping therapies.

## Material and methods

### Cell culture

Healthy control (HC (KM155)) and DMD patient cells (8036 and 6311) were kindly provided by Prof. Vincent Mouly (Institute de Myology) and have been described before [19,20,23]. Cells were maintained in skeletal muscle growth medium (Promocell C-23060), supplemented with 15% heat-inactivated foetal bovine serum (HI-FBS) and 50 µg/ml gentamycin. For differentiation of proliferating myoblasts to myotubes, medium was replaced with fusion media consisting of Dulbecco’s modified eagle medium (DMEM)-Glutamax, high-glucose (Gibco 61,965,059), supplemented with 2% HI-FBS and 1% penicillin-streptomycin (Gibco 15,140–122). Cells were maintained in humidified incubators at 37°C and 5% CO_2_. Cells were regularly tested for mycoplasma infection by use of the mycoalert (Lonza, LT07–318) kit. For cycloheximide (CHX) treatment experiments, cells were exposed to 100 µM CHX (Sigma Aldrich, C4859) for 6 hours, or DMSO as control.

### AON design

PMOs were designed to specifically target selected *DMD* exons, while exhibiting similar physical properties such as G/C content, number of nucleotides and melting temperature. For determination of the free energy of potential secondary structures (intramolecular and homodimer formation), RNAstructure 6.2 was used [24]. Each potential 24/25-mer AON targeting the 47 *DMD* exons outlined below was designed and analysed (~11.213 AONs in total). The average G/C content and melt temperature were calculated for the entire set, and AONs that deviated >1× standard deviation were excluded. Exons where less than 25 potential AONs remained were excluded from further consideration, as diversity of targeting sites would be too low. PMOs were synthesized and quality controlled in-house at Sarepta Therapeutics inc. Cambridge, MA, USA. PMOs were dissolved in sterile saline and diluted as stocks of 1 mM. Sequences and properties of all PMOs are listed in supplementary table 1.

### Electroporation

For delivery of PMOs to myoblasts cells, a Lonza Amaxa 4D nucleofector with accompanying X unit was utilized, as described in [25]. In brief, cells were trypsinized and resuspended in nucleofection P1 buffer at a concentration of 1 × 10^6^ cells per 20 µl of buffer. Then, 20 µl of P1 cell suspension was transferred to each well of the 16 well nucleocuvette, after which 1 µl of 1 mM PMO was added to the well, for a final concentration of 50 µM. Cells were electroporated in the X unit using program CM-137, and allowed to recover for 10 min at room temperature (RT). After recovery, cells were carefully resuspended in 200 µl growth medium and transferred to 6-well plates containing equilibrated growth medium. Cells were grown for at least 48 h before confluent cultures were allowed to differentiate to myotubes for 72 h as described.

### RNA isolation and cDNA synthesis

For RNA isolation, myotubes in each well were lysed in 500 µl Tri-sure lysis reagent, after which 200 µl chloroform was added and phases were separated by centrifugation at 16.200 relative centrifugal force (RCF) for 15 min at 4°C. The aqueous phase was transferred to 500 µl of 2-propanol and RNA was precipitated by centrifugation for 15 min at 4°C at 16.200 RCF. The pellet was washed twice with 70% ethanol, air-dried and resuspended in 25 µl RNAse-free milli-Q (MQ). RNA concentration and purity was determined using a ND-1000 Nanodrop (thermo-scientific) and provided as A_260_/A_230_ and A_260_/A_280_ ratios. For cDNA synthesis, 1 µg of total RNA was used using bioscript Tetro (bioline BIO-65050), according to the manufacturer’s instructions in a 20 µl reaction. Samples were diluted to 100 µl final volume with MQ.

### RT-PCR exon skipping analysis

RT-PCR analysis was used to determine *DMD* exon skipping efficiency. Ten percent of generated cDNA was used per reaction using specific sets of intron spanning primers (See supplementary table S2), and Dreamtaq polymerase (Thermo scientific EP0713). Reactions of 25 µl total consisted of 2.5 µl 10× reaction buffer (green), 0.2 µl DreamTaq polymerase (1 Unit), 1 µl 10 µM forward primer, 1 µl 10 µM reverse primer, 1 µl DNTP mix (10 µM each nucleotide) and 9.3 µl MQ. Amplification was performed in *T*-100 thermal cyclers (Bio-Rad) using the following parameters: 1: 95°C 2 min; 2: 95°C 30 sec; 3: 60°C 30 sec; 4: 72°C 45 sec; 5: Go to step 2, 34 additional cycles; 6: 72°C 5 min. A fraction of each sample was analysed using standard agarose TRIS-Borate-EDTA (TBE) gel-electrophoresis. DNA quantity in the PCR samples was assessed using Qubit dsDNA broad range reagent, measured in a Spectramax ID3 instrument. Samples were diluted to 0.2 ng/µl and analysed using an Agilent Femto Pulse with NGS separation gel. Peaks were called using the accompanying Prosize Data analysis software version 4.0.2.7. Skipping efficiency was calculated by determining the ratio of concentration (in nmole/L) of the skipped product over the total concentration (in nmole/L) of the sum of the full-length and skipped product. Statistical analysis was performed in Graphpad Prism Version 8.

### RT-qPCR analysis

For gene expression analysis, we used 2% of the generated cDNA samples described above per reaction. The RT-qPCR reaction consisted of 4 µl SensiMix 2× SYBR master mix (bioline QT605–05), 2 µl cDNA, 1 µl forward primer (10 µM) and 1 µl reverse primer (10 µM) (see supplementary table S2). Each sample/primer combination was measured in a technical triplicate. Samples were manually pipetted in 384-well plates (Framestar 480/384, 4ti-0381 4titude) and run in a CFX-384 Real-time PCR system (Bio-Rad). Cycling conditions were as follows: 1: 95°C 5:00; 2: 95°C 0:10; 3: 60°C 0:30 (plate read); 4: Go to step 2 39 additional times; 5: melt curve 60°C to 95°C, 0.5°C increase per cycle of 0:05. Data was analysed using the CFX-Maestro software version 2.0, with baseline and Cq value calculation set to ‘auto calculation mode’. CFX-Maestro was also used to determine run quality by studying the melt curves generated for each product, as well as internal QC functions. Expression values were normalized to the housekeeping genes *GAPDH* and *GUSB*, using the ΔΔCt method, after which statistical analysis and visualization was performed using Graphpad Prism Version 8.

### Dystrophin protein analysis

For dystrophin protein expression analysis, cells were electroporated with PMOs as described above. After 72 hours of differentiation, cells were harvested in Laemmli buffer (2% SDS, 10% glycerol in 60 mM TRIS-HCl [pH6.8]) and boiled to fully denature the protein samples. Samples were adjusted to 1 µg/µl after quantification with Pierce BCA protein assay kit (Thermofisher 23,227). Protein samples were further prepared for Wes analysis by diluting to a final concentration of 0.5 µg/µl with Wes standard pack 3 (Bio-Techne PS-ST03EZ-8), and run in a Wes 25-capillary electrophoresis system (Bio-Techne, SM-W008) as per the manufacturer’s instructions. Antibodies specific for dystrophin (Abcam, ab154168) and vinculin (Thermofisher, 700062) were diluted 1:100 in antibody diluent 2 for a duplex assay. QC and analysis of generated data was performed with Compass for Simple Western software (V6.1.0), after which data was exported and subsequently normalized in Excel 365 (Microsoft office).

## Supplementary Material

Supplemental Material

## Data Availability

The data that supports the findings of this study are included in the main and supplementary figures. Raw data can be supplied upon reasonable request to the corresponding author.
