# Supplementary material for: DMD antisense oligonucleotide mediated exon skipping efficiency correlates with flanking intron retention time and target position within the exon
Source: RNA Biol. 2023 Sep 4;20(1):693–702. doi: 10.1080/15476286.2023.2254041 (PMC10481881; doi:10.1080/15476286.2023.2254041)
Supplement: Supplemental Material [file KRNB_A_2254041_SM8312.zip › Supplementary_table1_PMO_sequences_LS.pdf]

PMO set 1

Flank type	#	PMO name	Reverse Complement	Tm°C	CG%	DGbimolecular	DGunimolecular	LEN	Frame	5'-Mid-3'	
Slow-Slow	1	Exon17-PMO-001	GAGTGGTGGTGACAGCCTGTGAAAT	71.4	52	-12.6	-2.3	25	Out		Exon 17: 6x
Slow-Slow	2	Exon17-PMO-037	TCTGTGTTAGTGATGGCTGAGTGGT	68.1	48	-12.8	-2.7	25	Out		
Slow-Slow	3	Exon17-PMO-115	CCCTTGTGGTCACCGTAGTTACTGT	68.1	52	-9	-0.8	25	Out		
Slow-Slow	4	Exon17-PMO-181	GTTCCTCTTGAGCATGCTTTACCAG	68	48	-14.8	-2.3	25	Out		
Slow-Slow	5	Exon17-PMO-261	ACAGTAATCTGCCTCTTCTTTTGGG	66.5	44	-9.7	-3.5	25	Out		
Slow-Slow	6	Exon17-PMO-281	TTCAGAATCCACAGTAATCTGCCTC	66.6	44	-5.4	-1.3	25	Out		
Slow-Slow	7	Exon21-PMO-001	GATCTGATAGCCGGTTGACTTCATC	67.9	48	-14.3	-0.3	25	Out		Exon 21: 7x
Slow-Slow	8	Exon21-PMO-013	GTTGAAGATCTGATAGCCGGTTGAC	67.9	48	-13.7	-0.3	25	Out		
Slow-Slow	9	Exon21-PMO-127	GTCCTTGTCCTTTCTCTTTCAGGGC	70	52	-10.8	-3.5	25	Out		
Slow-Slow	10	Exon21-PMO-146	GAACATGGGTCCTTGTCCTTTCTC	68	50	-11.6	-3.2	24	Out		
Slow-Slow	11	Exon21-PMO-197	TGTAAAGGCCACAAAGTCTGCATCC	70.8	48	-9.3	-2.6	25	Out		
Slow-Slow	12	Exon21-PMO-275	TCTGGCCTGCACATCAGAAAAGACT	71.1	48	-14.5	-3	25	Out		
Slow-Slow	13	Exon21-PMO-311	TGTCTGTAGCTCTTTCTCTCTGGCC	68.2	52	-5.9	0	25	Out		
Slow-Slow	14	Exon70-PMO-015	GCAAAGTCTCGAACATCTTCTCCTG	68.3	48	-5.1	0	25	Out		Exon 70: 5x
Slow-Slow	15	Exon70-PMO-031	GTACCTTGGCAAAGTCTCGAACATC	67.9	48	-6.2	0	25	Out		
Slow-Slow	16	Exon70-PMO-124	GGGGATGCTTCGCAAAATACCTTT	70.3	45.8	-7.7	-1.5	24	Out		
Slow-Slow	17	Exon70-PMO-207	TTGTCCCCCTCTAAGACAGTCTGCA	70.9	52	-9.6	-2	25	Out		
Slow-Slow	18	Exon70-PMO-221	TTCCATGTTGTCCCCCTCTAAGACA	70.4	48	-9.2	-1	25	Out		
Slow-Fast	19	Exon18-PMO-037	GCGAGTAATCCAGCTGTGAAGTTCA	69.5	48	-12.9	-1.9	25	Out		Exon 18: 5x
Slow-Fast	20	Exon18-PMO-065	AACACAGCTTCTGAGCGAGTAATCC	67.8	48	-10.1	-1.8	25	Out		
Slow-Fast	21	Exon18-PMO-101	GCAAATTCAGGACTCTGCAACACAG	70	48	-9	-1	25	Out		
Slow-Fast	22	Exon18-PMO-147	AGTTGCCTTCCTTCCGAAAGATTGC	71.7	48	-9.2	0	25	Out		
Slow-Fast	23	Exon18-PMO-167	AAGTCTGAGAAGTTGCCTTCCTTCC	67.9	48	-6.8	-1.9	25	Out		
Slow-Fast	24	Exon22-PMO-004	CGCATTGGTGGCAAAGTGTCAAAA	73.4	45.8	-11.2	-2.9	24	Out		Exon 22: 5x
Slow-Fast	25	Exon22-PMO-055	CTGATGGCACTCATGGTCTCCTGAT	71.6	52	-8.4	-1.7	25	Out		
Slow-Fast	26	Exon22-PMO-121	GAGAGTTTGGTTTCTGACTGCTGGA	68.8	48	-7.9	-2.4	25	Out		
Slow-Fast	27	Exon22-PMO-203	GCTCCATGATTTCATAGTCGGTGAC	68.7	48	-11.8	-2.7	25	Out		
Slow-Fast	28	Exon22-PMO-240	CAATTCCCCGAGTCTCTGCTCCAT	72.3	54.2	-8.4	-2.5	24	Out		
Slow-Fast	29	Exon50-PMO-019	CCTTCCACTCAGAGCTCAGATCTTC	67.8	52	-11.9	0	25	Out		Exon 50: 4x
Slow-Fast	30	Exon50-PMO-053	AAGTAAACGGTTTACCGCCTTCCAC	69.4	48	-14.3	-2.3	25	Out		
Slow-Fast	31	Exon50-PMO-095	CTGCTTTGCCCTCAGCTCTTGAAGT	71.6	52	-9.6	-1.3	25	Out		
Slow-Fast	32	Exon50-PMO-161	TCCAATAGTGGTCAGTCCAGGAGCT	70	52	-9.4	-2.8	25	Out		
Fast-Slow	33	Exon57-PMO-002	TCAGAACTGGCTTCCAAATGGGAC	71.2	50	-12.4	-2.4	24	Out		Exon 57: 6x
Fast-Slow	34	Exon57-PMO-053	AGAGAAAGGTGCAGACGCTTCCACT	71.2	52	-8.5	-2.8	25	Out		
Fast-Slow	35	Exon57-PMO-107	AGCTGTAGCCACACCAGAAGTTCCT	69.2	52	-9.6	-0.1	25	Out		
Fast-Slow	36	Exon57-PMO-173	ATAGGTGCCTGCCGGCTTAATTCAT	71.5	48	-14.1	-3.7	25	Out		
Fast-Slow	37	Exon57-PMO-217	AACTGCTGGAAAGTCGCCTCCAATA	71.3	48	-9.6	-3.3	25	Out		
Fast-Slow	38	Exon57-PMO-251	ACATCGTTCTGCTTCTGAACTGCTG	69.8	48	-9.7	-3	25	Out		
Fast-Slow	39	Exon65-PMO-005	ATGCAGCTGACAGGCTCAAGAGATC	71	52	-12.6	-3.9	25	Out		Exon 65: 7x
Fast-Slow	40	Exon65-PMO-077	CATTTTGCTTGAGGTTGTGCTGGTC	71.4	48	-7.4	-0.2	25	Out		
Fast-Slow	41	Exon65-PMO-111	GATATCCATGGGCTGGTCATTTTGC	71.7	48	-14.4	-0.9	25	Out		
Fast-Slow	42	Exon65-PMO-197	CCAGGCGGTCATAAATAGTGGTCAA	70.4	48	-8.7	-1.7	25	Out		
Fast-Slow	43	Exon65-PMO-243	GACCAAATTGTTGTGCTCTTGCTCC	70.6	48	-6.2	-1.1	25	Out		
Fast-Slow	44	Exon65-PMO-270	GAGAGGGACGTTGACCAAATTGTT	68.3	45.8	-8.7	-2	24	Out		
Fast-Slow	45	Exon65-PMO-301	AGACACATATCCACGCAGAGAGGGA	70.9	52	-5.2	-0.4	25	Out		
Fast-Slow	46	Exon67-PMO-004	GAACTTGCCACTTGCTTGAAAAGG	68.8	45.8	-5.1	-0.7	24	Out		Exon 67: 6x
Fast-Slow	47	Exon67-PMO-043	CGCTGGTCACAAAATCCTGTTGAAC	71.3	48	-7.2	-0.8	25	Out		
Fast-Slow	48	Exon67-PMO-103	ATAGAATCATGCAGAAGGAGGCCCA	71.3	48	-9	-0.9	25	Out		
Fast-Slow	49	Exon67-PMO-181	CCAAAGGATGCAACTTCACCCAACT	71.7	48	-6.9	-1	25	Out		
Fast-Slow	50	Exon67-PMO-233	GGACACTTGGCTCAATGTTACTGCC	70.5	52	-11.3	-2.2	25	Out		
Fast-Slow	51	Exon67-PMO-270	AAATTGGAAGCAGCTCCGGACACT	71.6	50	-15	-3	24	Out		
Fast-Fast	52	Exon55-PMO-010	TTCCAAAGCAGCCTCTCGCTCACT	73.1	54.2	-6.1	-1.7	24	Out		Exon 55: 7x
Fast-Fast	53	Exon55-PMO-028	ATGAGTTTCTTCCAAAGCAGCCTC	67.8	45.8	-6.3	-1	24	Out		
Fast-Fast	54	Exon55-PMO-075	CCAGGGGGAACTGTTGCAGTAATCT	70.8	52	-14.8	-2.8	25	Out		
Fast-Fast	55	Exon55-PMO-117	AGGCAAGAAACTTTTCCAGGTCCAG	70.1	48	-8.1	-1.1	25	Out		
Fast-Fast	56	Exon55-PMO-155	GTTTCAGCTTCTGTAAGCCAGGCAA	70.2	48	-11.8	-3.1	25	Out		
Fast-Fast	57	Exon55-PMO-245	AGGAGCCTTTCCTTACGGGTAGCAT	70.2	52	-11.1	-3.7	25	Out		
Fast-Fast	58	Exon55-PMO-309	TCATCAGCTCTTTTACTCCCTTGGA	68	44	-5.9	0	25	Out		
Fast-Fast	59	Exon51-PMO-027	CACAGGTTGTGTCACCAGAGTAACA	67.4	48	-11.8	-1.9	25	Out		Exon 51: 8x
Fast-Fast	60	Exon51-PMO-043	TTAGTAACCACAGGTTGTGTCACCA	66.5	44	-12.6	-2.1	25	Out		
Fast-Fast	61	Exon51-PMO-147	TACCTCCAACATCAAGGAAGATGGC	70	48	-9	-3.3	25	Out		
Fast-Fast	62	Exon51-PMO-229	GTAAGTTCTGTCCAAGCCCGGTTGA	71.7	52	-9.4	-0.2	25	Out		
Fast-Fast	63	Exon51-PMO-281	CTTGATCAAGCAGAGAAAGCCAGTC	68.4	48	-12.8	-1.1	25	Out		
Fast-Fast	64	Exon51-PMO-337	CCCACCATCACCCTCTGTGATTTTA	70.6	48	-9.2	-3	25	Out		
Fast-Fast	65	Exon51-PMO-365	TATCCTCAAGGTCACCCACCATCAC	71	52	-7	-1.6	25	Out		
Fast-Fast	66	Exon51-PMO-393	GATCATCTCGTTGATATCCTCAAGG	65.9	44	-9.5	-2	25	Out		
Fast-Fast	67	Exon59-PMO-021	AGTGACATTCTGGGCTCTCTCCTCA	70.5	52	-9.6	-2.7	25	Out		Exon 59: 8x
Fast-Fast	68	Exon59-PMO-055	TGCTTTCGTAGAAGCCGAGTGACAT	70.7	48	-10.4	-3.1	25	Out		
Fast-Fast	69	Exon59-PMO-093	AGTATTGACCTCCTCAGCCTGCTTT	68.2	48	-5.7	-1	25	Out		
Fast-Fast	70	Exon59-PMO-193	TCTATTTTTCTCTGCCAGTCAGCGG	70.2	48	-7.2	-2.6	25	Out		
Fast-Fast	71	Exon59-PMO-247	AGTTCCCGGAGTCTTTCAAGGGTCT	70.8	52	-12.1	-3.4	25	Out		
Fast-Fast	72	Exon59-PMO-373	CAGGATCCCTTGATCACCTCAGCTT	71.4	52	-13.2	-2.8	25	Out		
Fast-Fast	73	Exon59-PMO-447	TTGGAGAGAGTCAATGAGGAGATCG	68.9	48	-7.4	-0.6	25	Out		
Fast-Fast	74	Exon59-PMO-489	CTTGACTTTCTCGAGGTGATCTTGG	68.2	48	-15	-3	25	Out		

#	Skipping efficiency N=1		Skiping efficiency N=2		Average	Stdev
1	Exon17-PMO-001	**45.62%**	Exon17-PMO-001	**57.67%**	51.64%	0.09
2	Exon17-PMO-037	**46.21%**	Exon17-PMO-037	**49.14%**	47.68%	0.02
3	Exon17-PMO-115	**66.95%**	Exon17-PMO-115	**76.48%**	71.71%	0.07
4	Exon17-PMO-181	**55.15%**	Exon17-PMO-181	**63.81%**	59.48%	0.06
5	Exon17-PMO-261	**27.25%**	Exon17-PMO-261	**20.31%**	23.78%	0.05
6	Exon17-PMO-281	**0.00%**	Exon17-PMO-281	**3.05%**	1.52%	0.02
7	Exon21-PMO-001	**58.04%**	Exon21-PMO-001	**38.65%**	48.34%	0.14
8	Exon21-PMO-013	**66.89%**	Exon21-PMO-013	**38.60%**	52.74%	0.20
9	Exon21-PMO-127	**1.53%**	Exon21-PMO-127	**2.67%**	2.10%	0.01
10	Exon21-PMO-146	**6.19%**	Exon21-PMO-146	**7.41%**	6.80%	0.01
11	Exon21-PMO-197	**4.37%**	Exon21-PMO-197	**6.21%**	5.29%	0.01
12	Exon21-PMO-275	**1.06%**	Exon21-PMO-275	**2.10%**	1.58%	0.01
13	Exon21-PMO-311	**26.18%**	Exon21-PMO-311	**14.06%**	20.12%	0.09
14	Exon70-PMO-015	**24.13%**	Exon70-PMO-015	**15.97%**	20.05%	0.06
15	Exon70-PMO-031	**8.97%**	Exon70-PMO-031	**36.80%**	22.89%	0.20
16	Exon70-PMO-124	**30.69%**	Exon70-PMO-124	**32.10%**	31.40%	0.01
17	Exon70-PMO-207	**0.58%**	Exon70-PMO-207	**1.10%**	0.84%	0.00
18	Exon70-PMO-221	**12.89%**	Exon70-PMO-221	**11.70%**	12.30%	0.01
19	Exon18-PMO-037	**63.31%**	Exon18-PMO-037	**35.32%**	49.32%	0.20
20	Exon18-PMO-065	**46.98%**	Exon18-PMO-065	**38.39%**	42.68%	0.06
21	Exon18-PMO-101	**42.64%**	Exon18-PMO-101	**57.47%**	50.05%	0.10
22	Exon18-PMO-147	**42.90%**	Exon18-PMO-147	**60.02%**	51.46%	0.12
23	Exon18-PMO-167	**32.36%**	Exon18-PMO-167	**52.46%**	42.41%	0.14
24	Exon22-PMO-004	**0.00%**	Exon22-PMO-004	**2.46%**	1.23%	0.02
25	Exon22-PMO-055	**92.00%**	Exon22-PMO-055	**59.97%**	75.99%	0.23
26	Exon22-PMO-121	**77.62%**	Exon22-PMO-121	**50.16%**	63.89%	0.19
27	Exon22-PMO-203	**83.30%**	Exon22-PMO-203	**64.41%**	73.85%	0.13
28	Exon22-PMO-240	**47.88%**	Exon22-PMO-240	**32.27%**	40.07%	0.11
29	Exon50-PMO-019	**94.16%**	Exon50-PMO-019	**58.58%**	76.37%	0.25
30	Exon50-PMO-053	**64.69%**	Exon50-PMO-053	**40.99%**	52.84%	0.17
31	Exon50-PMO-095	**57.90%**	Exon50-PMO-095	**25.43%**	41.66%	0.23
32	Exon50-PMO-161	**0.00%**	Exon50-PMO-161	**7.95%**	3.98%	0.06
33	Exon57-PMO-002	**23.56%**	Exon57-PMO-002	**15.17%**	19.37%	0.06
34	Exon57-PMO-053	**38.97%**	Exon57-PMO-053	**15.50%**	27.23%	0.17
35	Exon57-PMO-107	**2.50%**	Exon57-PMO-107	**1.95%**	2.23%	0.00
36	Exon57-PMO-173	**0.69%**	Exon57-PMO-173	**0.62%**	0.65%	0.00
37	Exon57-PMO-217	**0.82%**	Exon57-PMO-217	**0.55%**	0.68%	0.00
38	Exon57-PMO-251	**0.61%**	Exon57-PMO-251	**0.66%**	0.63%	0.00
39	Exon65-PMO-005	**0.00%**	Exon65-PMO-005	**0.00%**	0.00%	0.00
40	Exon65-PMO-077	**0.00%**	Exon65-PMO-077	**0.00%**	0.00%	0.00
41	Exon65-PMO-111	**0.00%**	Exon65-PMO-111	**0.00%**	0.00%	0.00
42	Exon65-PMO-197	**0.00%**	Exon65-PMO-197	**0.00%**	0.00%	0.00
43	Exon65-PMO-243	**0.00%**	Exon65-PMO-243	**0.00%**	0.00%	0.00
44	Exon65-PMO-270	**0.00%**	Exon65-PMO-270	**0.00%**	0.00%	0.00
45	Exon65-PMO-301	**0.00%**	Exon65-PMO-301	**0.00%**	0.00%	0.00
46	Exon67-PMO-004	**7.89%**	Exon67-PMO-004	**6.57%**	7.23%	0.01
47	Exon67-PMO-043	**7.78%**	Exon67-PMO-043	**5.18%**	6.48%	0.02
48	Exon67-PMO-103	**5.20%**	Exon67-PMO-103	**1.35%**	3.27%	0.03
49	Exon67-PMO-181	**1.62%**	Exon67-PMO-181	**0.70%**	1.16%	0.01
50	Exon67-PMO-233	**0.00%**	Exon67-PMO-233	**0.00%**	0.00%	0.00
51	Exon67-PMO-270	**11.62%**	Exon67-PMO-270	**23.38%**	17.50%	0.08
52	Exon55-PMO-010	**29.68%**	Exon55-PMO-010	**35.92%**	32.80%	0.04
53	Exon55-PMO-028	**86.07%**	Exon55-PMO-028	**55.50%**	70.79%	0.22
54	Exon55-PMO-075	**22.74%**	Exon55-PMO-075	**13.52%**	18.13%	0.07
55	Exon55-PMO-117	**68.12%**	Exon55-PMO-117	**58.64%**	63.38%	0.07
56	Exon55-PMO-155	**4.18%**	Exon55-PMO-155	**6.35%**	5.26%	0.02
57	Exon55-PMO-245	**4.94%**	Exon55-PMO-245	**4.81%**	4.88%	0.00
58	Exon55-PMO-309	**0.00%**	Exon55-PMO-309	**0.69%**	0.34%	0.00
59	Exon51-PMO-027	**15.83%**	Exon51-PMO-027	**11.29%**	13.56%	0.03
60	Exon51-PMO-043	**6.87%**	Exon51-PMO-043	**1.66%**	4.27%	0.04
61	Exon51-PMO-147	**14.45%**	Exon51-PMO-147	**9.90%**	12.17%	0.03
62	Exon51-PMO-229	**2.69%**	Exon51-PMO-229	**5.17%**	3.93%	0.02
63	Exon51-PMO-281	**7.74%**	Exon51-PMO-281	**6.39%**	7.07%	0.01
64	Exon51-PMO-337	**0.00%**	Exon51-PMO-337	**0.00%**	0.00%	0.00
65	Exon51-PMO-365	**0.21%**	Exon51-PMO-365	**0.00%**	0.10%	0.00
66	Exon51-PMO-393	**0.92%**	Exon51-PMO-393	**0.00%**	0.46%	0.01
67	Exon59-PMO-021	**0.00%**	Exon59-PMO-021	**0.42%**	0.21%	0.00
68	Exon59-PMO-055	**0.50%**	Exon59-PMO-055	**0.65%**	0.57%	0.00
69	Exon59-PMO-093	**0.00%**	Exon59-PMO-093	**0.00%**	0.00%	0.00
70	Exon59-PMO-193	**0.00%**	Exon59-PMO-193	**0.40%**	0.20%	0.00
71	Exon59-PMO-247	**0.35%**	Exon59-PMO-247	**0.00%**	0.17%	0.00
72	Exon59-PMO-373	**0.00%**	Exon59-PMO-373	**0.00%**	0.00%	0.00
73	Exon59-PMO-447	**0.00%**	Exon59-PMO-447	**0.00%**	0.00%	0.00
74	Exon59-PMO-489	**0.00%**	Exon59-PMO-489	**0.00%**	0.00%	0.00

PMO set 2

Flank type		PMO name	Reverse Complement	Tm°C	CG%	DGbimolecular	DGunimolecular	LEN	Frame	5'-Mid-3'						
Slow-Fast	1	Exon18-PMO-037	GCGAGTAATCCAGCTGTGAAGTTCA	69.5	48	-12.9	-1.9	25	Out		Exon18-PMO-037	**62.82%**	Exon18-PMO-037	**59.23%**	61.02%	0.03
Slow-Fast	2	Exon18-PMO-047	TCTGAGCGAGTAATCCAGCTGTGAA	70.2	48	-8.6	-0.7	25	Out		Exon18-PMO-047	**59.84%**	Exon18-PMO-047	**62.66%**	61.25%	0.02
Slow-Fast	3	Exon18-PMO-055	AGCTTCTGAGCGAGTAATCCAGCTG	69.8	52	-11.3	-2	25	Out		Exon18-PMO-055	**59.84%**	Exon18-PMO-055	**62.16%**	61.00%	0.02
Slow-Fast	4	Exon18-PMO-065	AACACAGCTTCTGAGCGAGTAATCC	67.8	48	-10.1	-1.8	25	Out		Exon18-PMO-065	**55.12%**	Exon18-PMO-065	**56.94%**	56.03%	0.01
Slow-Fast	5	Exon18-PMO-139	GCCTTCCTTCCGAAAGATTGCAAAT	71.4	44	-10.6	-0.1	25	Out		Exon18-PMO-139	**53.96%**	Exon18-PMO-139	**53.69%**	53.82%	0.00
Slow-Fast	6	Exon18-PMO-147	AGTTGCCTTCCTTCCGAAAGATTGC	71.7	48	-9.2	0	25	Out		Exon18-PMO-147	**56.30%**	Exon18-PMO-147	**54.75%**	55.53%	0.01
Slow-Fast	7	Exon18-PMO-157	TGAGAAGTTGCCTTCCTTCCGAAAG	71.1	48	-9.6	-2	25	Out		Exon18-PMO-157	**46.72%**	Exon18-PMO-157	**47.56%**	47.14%	0.01
Slow-Fast	8	Exon18-PMO-167	AAGTCTGAGAAGTTGCCTTCCTTCC	67.9	48	-6.8	-1.9	25	Out		Exon18-PMO-167	**53.09%**	Exon18-PMO-167	**51.78%**	52.44%	0.01
Slow-Fast	9	Exon22-PMO-018	CTGATAGCGCATTGGTGGCAAAGT	71.8	50	-12	-0.8	24	Out		Exon22-PMO-018	**0.71%**	Exon22-PMO-018	**2.16%**	1.43%	0.01
Slow-Fast	10	Exon22-PMO-031	ATGGTCTCCTGATAGCGCATTGGTG	72.7	52	-8.7	-0.5	25	Out		Exon22-PMO-031	**88.98%**	Exon22-PMO-031	**91.05%**	90.01%	0.01
Slow-Fast	11	Exon22-PMO-041	CACTCATGGTCTCCTGATAGCGCAT	71.3	52	-8.7	-0.3	25	Out		Exon22-PMO-041	**71.33%**	Exon22-PMO-041	**86.86%**	79.09%	0.11
Slow-Fast	12	Exon22-PMO-055	CTGATGGCACTCATGGTCTCCTGAT	71.6	52	-8.4	-1.7	25	Out		Exon22-PMO-055	**65.63%**	Exon22-PMO-055	**61.34%**	63.49%	0.03
Slow-Fast	13	Exon22-PMO-203	GCTCCATGATTTCATAGTCGGTGAC	68.7	48	-11.8	-2.7	25	Out		Exon22-PMO-203	**79.14%**	Exon22-PMO-203	**69.03%**	74.08%	0.07
Slow-Fast	14	Exon22-PMO-213	TCTCTGCTCCATGATTTCATAGTCG	67.5	44	-5.3	0	25	Out		Exon22-PMO-213	**59.27%**	Exon22-PMO-213	**59.42%**	59.34%	0.00
Slow-Fast	15	Exon22-PMO-223	CCGAGTCTCTGCTCCATGATTTCAT	70.4	48	-9.7	-1.6	25	Out		Exon22-PMO-223	**40.32%**	Exon22-PMO-223	**41.56%**	40.94%	0.01
Slow-Fast	16	Exon22-PMO-233	ATTCCCCGAGTCTCTGCTCCATGAT	72.6	52	-6.9	-0.5	25	Out		Exon22-PMO-233	**27.12%**	Exon22-PMO-233	**37.61%**	32.36%	0.07
Slow-Fast	17	Exon53-PMO-023	CTTGTACTTCATCCCACTGATTCTG	66.8	48	-4.2	0	25	Out		Exon53-PMO-023	**5.48%**	Exon53-PMO-023	**10.8%**	8.16%	0.04
Slow-Fast	18	Exon53-PMO-033	GTGTTCTTGTACTTCATCCCACTGA	68.3	48	-7	-0.6	25	Out		Exon53-PMO-033	**0.00%**	Exon53-PMO-033	**7.4%**	3.70%	0.05
Slow-Fast	19	Exon53-PMO-043	TGAAGGTGTTCTTGTACTTCATCCC	72.4	48	-8.4	0	25	Out		Exon53-PMO-043	**12.27%**	Exon53-PMO-043	**11.1%**	11.67%	0.01
Slow-Fast	20	Exon53-PMO-055	CGGTTCTGAAGGTGTTCTTGTACTT	70.8	45.8	-8.4	0	25	Out		Exon53-PMO-055	**9.19%**	Exon53-PMO-055	**4.0%**	6.59%	0.04
Slow-Fast	21	Exon53-PMO-279	CTCCTTCCATGACTCAAGCTTGGCT	71.5	52	-10.4	-0.6	25	Out		Exon53-PMO-279	**0.78%**	Exon53-PMO-279	**2.7%**	1.76%	0.01
Slow-Fast	22	Exon53-PMO-299	TATAGGGACCCTCCTTCCATGACTC	68.2	52	-9.5	-1	25	Out		Exon53-PMO-299	**2.29%**	Exon53-PMO-299	**3.0%**	2.63%	0.00
Slow-Fast	23	Exon53-PMO-309	TACTGTATAGGGACCCTCCTTCCAT	65.8	48	-9.5	-0.6	25	Out		Exon53-PMO-309	**1.57%**	Exon53-PMO-309	**1.9%**	1.74%	0.00
Slow-Fast	24	Exon53-PMO-321	TGCATCTACTGTATAGGGACCCTCC	67.2	52	-10.1	-0.8	25	Out		Exon53-PMO-321	**0.00%**	Exon53-PMO-321	**1.1%**	0.55%	0.01
Slow-Fast	25	Exon10-PMO-001	ACTTGTCTTCAGGAGCTTCCAAATG	67.7	44	-7.5	-0.8	25	In		Exon10-PMO-001	**0.00%**	Exon10-PMO-001	**0.5%**	0.26%	0.00
Slow-Fast	26	Exon10-PMO-011	AAATGACTTGTCTTCAGGAGCTTCC	66.8	44	-7.5	-0.5	25	In		Exon10-PMO-011	**0.00%**	Exon10-PMO-011	**0.0%**	0.00%	0.00
Slow-Fast	27	Exon10-PMO-021	CTGCCAAATGACTTGTCTTCAGGAG	69.1	48	-4.9	-0.4	25	In		Exon10-PMO-021	**0.00%**	Exon10-PMO-021	**0.6%**	0.30%	0.00
Slow-Fast	28	Exon10-PMO-047	CTCCATCAATGAACTGCCAAATGAC	69.5	44	-4.8	0	25	In		Exon10-PMO-047	**0.00%**	Exon10-PMO-047	**0.0%**	0.00%	0.00
Slow-Fast	29	Exon10-PMO-233	CTCTCCTTGTGCTTGCAATGTGTCC	71.9	52	-6.8	-0.5	25	In		Exon10-PMO-233	**0.00%**	Exon10-PMO-233	**0.1%**	0.06%	0.00
Slow-Fast	30	Exon10-PMO-243	GAAATCTCTCCTTGTGCTTGCAATG	68.9	44	-6.8	-0.5	25	In		Exon10-PMO-243	**0.00%**	Exon10-PMO-243	**0.0%**	0.00%	0.00
Slow-Fast	31	Exon10-PMO-301	ACTGGTCTTTCACCACTTCCACATC	68.5	48	-5	-1.6	25	In		Exon10-PMO-301	**0.00%**	Exon10-PMO-301	**0.0%**	0.00%	0.00
Slow-Fast	32	Exon10-PMO-311	ATGAAACTGGTCTTTCACCACTTCC	67.4	44	-8.1	-2.1	25	In		Exon10-PMO-311	**0.00%**	Exon10-PMO-311	**0.0%**	0.00%	0.00
Slow-Fast	33	Exon14-PMO-001	TGTTTGCCCATCGATCTCCCAATAC	72.2	48	-6	0	25	In		Exon14-PMO-001	**8.60%**	Exon14-PMO-001	**23.2%**	15.89%	0.10
Slow-Fast	34	Exon14-PMO-013	TACAGATGTTTGCCCATCGATCTCC	70.8	48	-6.7	-1.2	25	In		Exon14-PMO-013	**1.14%**	Exon14-PMO-013	**2.5%**	1.80%	0.01
Slow-Fast	35	Exon14-PMO-023	CCATCTACAGATGTTTGCCCATCGA	71.7	48	-8.1	-1.2	25	In		Exon14-PMO-023	**40.37%**	Exon14-PMO-023	**44.3%**	42.33%	0.03
Slow-Fast	36	Exon14-PMO-033	TCTGTCCATCTACAGATGTTTGCCC	69.2	48	-8.1	-2	25	In		Exon14-PMO-033	**18.89%**	Exon14-PMO-033	**38.2%**	28.56%	0.14
Slow-Fast	37	Exon14-PMO-125	ACGTTGCCATTTGAGAAGGATGTCT	69.7	44	-4.4	0	25	In		Exon14-PMO-125	**1.09%**	Exon14-PMO-125	**3.7%**	2.41%	0.02
Slow-Fast	38	Exon14-PMO-135	GTAAGACGTTGCCATTTGAGAAGGA	68.2	44	-5.9	0	25	In		Exon14-PMO-135	**0.64%**	Exon14-PMO-135	**1.2%**	0.92%	0.00
Slow-Fast	39	Exon14-PMO-145	CTTCAGTAAGACGTTGCCATTTGAG	66.9	44	-5.9	0	25	In		Exon14-PMO-145	**0.62%**	Exon14-PMO-145	**0.6%**	0.63%	0.00
Slow-Fast	40	Exon14-PMO-155	CTGTTCTTCAGTAAGACGTTGCCAT	66.5	44	-6.3	-1	25	In		Exon14-PMO-155	**13.16%**	Exon14-PMO-155	**32.3%**	22.73%	0.14
Slow-Fast	41	Exon42-PMO-001	TCATCGTTTCTTCACGGACAGTGTG	71.3	48	-11.1	-1.3	25	In		Exon42-PMO-001	**95.97%**	Exon42-PMO-001	**88.61%**	92.29%	0.05
Slow-Fast	42	Exon42-PMO-011	CACCATCATCGTTTCTTCACGGACA	72.8	48	-4.5	-0.5	25	In		Exon42-PMO-011	**95.26%**	Exon42-PMO-011	**81.63%**	88.44%	0.10
Slow-Fast	43	Exon42-PMO-021	GTCATCACCATCATCGTTTCTTCAC	67.8	44	-3	0	25	In		Exon42-PMO-021	**88.19%**	Exon42-PMO-021	**61.26%**	74.73%	0.19
Slow-Fast	44	Exon42-PMO-031	CTTCAGTCATCACCATCATCGTTTC	67.9	44	-3	0	25	In		Exon42-PMO-031	**96.34%**	Exon42-PMO-031	**70.20%**	83.27%	0.18
Slow-Fast	45	Exon42-PMO-257	AGCACAGAGGTCAGGAGCATTGAGA	71.5	52	-3.4	0	25	In		Exon42-PMO-257	**6.12%**	Exon42-PMO-257	**11.40%**	8.76%	0.04
Slow-Fast	46	Exon42-PMO-267	TCCTTAGCACAGAGGTCAGGAGCAT	70.2	52	-6.2	-0.4	25	In		Exon42-PMO-267	**6.06%**	Exon42-PMO-267	**7.31%**	6.69%	0.01
Slow-Fast	47	Exon42-PMO-277	CAAAGTCCTTAGCACAGAGGTCAGG	68.2	52	-4.4	-0.4	25	In		Exon42-PMO-277	**11.77%**	Exon42-PMO-277	**14.66%**	13.21%	0.02
Slow-Fast	48	Exon42-PMO-287	ATCTTCAAAGTCCTTAGCACAGAGG	65	44	-6.5	-0.4	25	In		Exon42-PMO-287	**9.89%**	Exon42-PMO-287	**19.91%**	14.90%	0.07
Fast-Slow	49	Exon52-PMO-001	TCTGTTCCAAATCCTGCATTGTTGC	71.7	44	-5.1	-1.4	25	Out		Exon52-PMO-001	**44.03%**	Exon52-PMO-001	**54.0%**	49.02%	0.07
Fast-Slow	50	Exon52-PMO-012	CGCCTCTGTTCCAAATCCTGCATT	72.7	50	-4.8	0	24	Out		Exon52-PMO-012	**10.12%**	Exon52-PMO-012	**21.7%**	15.92%	0.08
Fast-Slow	51	Exon52-PMO-053	AATGAGTTCTTCCAACTGGGGACGC	72.7	52	-6.1	-1	25	Out		Exon52-PMO-053	**16.53%**	Exon52-PMO-053	**46.5%**	31.51%	0.21
Fast-Slow	52	Exon52-PMO-063	GCGGTAATGAGTTCTTCCAACTGGG	71.1	52	-6.2	-1	25	Out		Exon52-PMO-063	**52.22%**	Exon52-PMO-063	**54.7%**	53.44%	0.02
Fast-Slow	53	Exon52-PMO-151	TTCTAGCCTCTTGATTGCTGGTCTT	67.3	44	-4.9	-0.7	25	Out		Exon52-PMO-151	**4.71%**	Exon52-PMO-151	**4.8%**	4.76%	0.00
Fast-Slow	54	Exon52-PMO-161	GATTGTTCTAGCCTCTTGATTGCTG	66.2	44	-4.9	-1	25	Out		Exon52-PMO-161	**3.31%**	Exon52-PMO-161	**6.1%**	4.72%	0.02
Fast-Slow	55	Exon52-PMO-175	CCGTAATGATTGTTCTAGCCTCTTG	65.8	44	-3.6	0	25	Out		Exon52-PMO-175	**2.98%**	Exon52-PMO-175	**3.3%**	3.14%	0.00
Fast-Slow	56	Exon52-PMO-185	TCGATCCGTAATGATTGTTCTAGCC	67.9	44	-5.5	0	25	Out		Exon52-PMO-185	**3.62%**	Exon52-PMO-185	**4.7%**	4.15%	0.01
Fast-Slow	57	Exon57-PMO-002	TCAGAACTGGCTTCCAAATGGGAC	71.2	50	-12.4	-2.4	24	Out		Exon57-PMO-002	**8.77%**	Exon57-PMO-002	**41.52%**	25.14%	0.23
Fast-Slow	58	Exon57-PMO-011	CACTGGTCAGAACTGGCTTCCAAAT	70.2	48	-6.5	-0.3	25	Out		Exon57-PMO-011	**3.11%**	Exon57-PMO-011	**8.26%**	5.69%	0.04
Fast-Slow	59	Exon57-PMO-053	AGAGAAAGGTGCAGACGCTTCCACT	71.2	52	-8.5	-2.8	25	Out		Exon57-PMO-053	**13.25%**	Exon57-PMO-053	**42.16%**	27.71%	0.20
Fast-Slow	60	Exon57-PMO-079	CAGAAGTTCCTGCAGAGAAAGGTGC	70.2	52	-10.2	-0.6	25	Out		Exon57-PMO-079	**14.69%**	Exon57-PMO-079	**43.19%**	28.94%	0.20
Fast-Slow	61	Exon57-PMO-217	AACTGCTGGAAAGTCGCCTCCAATA	71.3	48	-9.6	-3.3	25	Out		Exon57-PMO-217	**0.88%**	Exon57-PMO-217	**2.90%**	1.89%	0.01
Fast-Slow	62	Exon57-PMO-235	CTGCTTCTGAACTGCTGGAAAGTCG	71.1	52	-5.4	0	25	Out		Exon57-PMO-235	**2.03%**	Exon57-PMO-235	**3.84%**	2.94%	0.01
Fast-Slow	63	Exon57-PMO-251	ACATCGTTCTGCTTCTGAACTGCTG	69.8	48	-9.7	-3	25	Out		Exon57-PMO-251	**0.55%**	Exon57-PMO-251	**1.34%**	0.95%	0.01
Fast-Slow	64	Exon57-PMO-265	CCTATGTACATCGTTCTGCTTCTGA	65.5	44	-8.2	0	25	Out		Exon57-PMO-265	**1.53%**	Exon57-PMO-265	**4.43%**	2.98%	0.02
Fast-Slow	65	Exon65-PMO-005	ATGCAGCTGACAGGCTCAAGAGATC	71	52	-12.6	-3.9	25	Out		Exon65-PMO-005	**0.00%**	Exon65-PMO-005	**0.0%**	0.00%	0.00
Fast-Slow	66	Exon65-PMO-015	ATCACATGCAGCTGACAGGCTCAAG	72.6	52	-10.9	-1.5	25	Out		Exon65-PMO-015	**0.00%**	Exon65-PMO-015	**0.0%**	0.00%	0.00
Fast-Slow	67	Exon65-PMO-077	CATTTTGCTTGAGGTTGTGCTGGTC	71.4	48	-7.4	-0.2	25	Out		Exon65-PMO-077	**0.00%**	Exon65-PMO-077	**0.0%**	0.00%	0.00
Fast-Slow	68	Exon65-PMO-087	CTGGTCATTTTGCTTGAGGTTGTGC	71.4	48	-3.3	0	25	Out		Exon65-PMO-087	**0.00%**	Exon65-PMO-087	**0.0%**	0.00%	0.00
Fast-Slow	69	Exon65-PMO-301	AGACACATATCCACGCAGAGAGGGA	70.9	52	-5.2	-0.4	25	Out		Exon65-PMO-301	**0.00%**	Exon65-PMO-301	**0.0%**	0.00%	0.00
Fast-Slow	70	Exon65-PMO-315	CCAGTTCAGACACATATCCACGCAG	70.7	52	-3.4	0	25	Out		Exon65-PMO-315	**0.00%**	Exon65-PMO-315	**0.0%**	0.00%	0.00
Fast-Slow	71	Exon65-PMO-331	TTCAGCAGCCAGTTCAGACACATAT	68	44	-4.1	0	25	Out		Exon65-PMO-331	**0.00%**	Exon65-PMO-331	**0.0%**	0.00%	0.00
Fast-Slow	72	Exon65-PMO-347	CATAAACATTCAGCAGCCAGTTCAG	67.7	44	-4.1	0	25	Out		Exon65-PMO-347	**0.00%**	Exon65-PMO-347	**0.0%**	0.00%	0.00
Fast-Slow	73	Exon67-PMO-004	GAACTTGCCACTTGCTTGAAAAGG	68.8	45.8	-5.1	-0.7	24	Out		Exon67-PMO-004	**20.06%**	Exon67-PMO-004	**33.50%**	26.78%	0.10
Fast-Slow	74	Exon67-PMO-015	TCCTGTTGAACTTGCCACTTGCTTG	72.1	48	-5.3	-0.3	25	Out		Exon67-PMO-015	**24.44%**	Exon67-PMO-015	**43.45%**	33.94%	0.13
Fast-Slow	75	Exon67-PMO-029	CACAAAATCCTGTTGAACTTGCCAC	69.4	44	-4.7	-0.1	25	Out		Exon67-PMO-029	**33.21%**	Exon67-PMO-029	**39.14%**	36.18%	0.04
Fast-Slow	76	Exon67-PMO-043	CGCTGGTCACAAAATCCTGTTGAAC	71.3	48	-7.2	-0.8	25	Out		Exon67-PMO-043	**10.64%**	Exon67-PMO-043	**23.40%**	17.02%	0.09
Fast-Slow	77	Exon67-PMO-216	CTCAATGTTACTGCCCCCAAAGGA	70.7	50	-5.2	0	24	Out		Exon67-PMO-216	**3.38%**	Exon67-PMO-216	**8.29%**	5.84%	0.03
Fast-Slow	78	Exon67-PMO-233	GGACACTTGGCTCAATGTTACTGCC	70.5	52	-11.3	-2.2	25	Out		Exon67-PMO-233	**1.04%**	Exon67-PMO-233	**0.67%**	0.85%	0.00
Fast-Slow	79	Exon67-PMO-243	GCTCCGGACACTTGGCTCAATGTTA	72.7	52	-9.9	-0.6	25	Out		Exon67-PMO-243	**3.04%**	Exon67-PMO-243	**4.44%**	3.74%	0.01
Fast-Slow	80	Exon67-PMO-270	AAATTGGAAGCAGCTCCGGACACT	71.6	50	-15	-3	24	Out		Exon67-PMO-270	**16.18%**	Exon67-PMO-270	**40.57%**	28.38%	0.17
Fast-Slow	81	Exon09-PMO-001	ATCCCTGTGCTAGACTGACCGTGAT	70	52	-3.8	0	25	In		Exon09-PMO-001	**93.76%**	Exon09-PMO-001	**67.61%**	80.69%	0.18
Fast-Slow	82	Exon09-PMO-011	CTCATATCCCTGTGCTAGACTGACC	66.2	52	-3.8	0	25	In		Exon09-PMO-011	**83.38%**	Exon09-PMO-011	**72.21%**	77.79%	0.08
Fast-Slow	83	Exon09-PMO-059	TCGAGGCTTAGGGGAAGAAGTTCTC	69.5	52	-7.5	-0.4	25	In		Exon09-PMO-059	**62.60%**	Exon09-PMO-059	**50.53%**	56.56%	0.09
Fast-Slow	84	Exon09-PMO-069	TTGAATCGAGGCTTAGGGGAAGAAG	70	48	-5.2	-0.1	25	In		Exon09-PMO-069	**58.02%**	Exon09-PMO-069	**41.43%**	49.73%	0.12
Fast-Slow	85	Exon09-PMO-141	GTGGTGACATAAGCAGCCTGTGTGT	70.4	52	-6.4	-0.2	25	In		Exon09-PMO-141	**84.83%**	Exon09-PMO-141	**60.27%**	72.55%	0.17
Fast-Slow	86	Exon09-PMO-161	AGGGTCAGAGGTGGTGACATAAGCA	70.8	52	-6.4	-1.5	25	In		Exon09-PMO-161	**72.76%**	Exon09-PMO-161	**52.77%**	62.77%	0.14
Fast-Slow	87	Exon09-PMO-169	GTGTAGGGTCAGAGGTGGTGACATA	67.3	52	-6.4	-1.5	25	In		Exon09-PMO-169	**66.58%**	Exon09-PMO-169	**53.70%**	60.14%	0.09
Fast-Slow	88	Exon09-PMO-209	CTGTGAAGGAAATGGGCTCCGTGTA	72.6	52	-4.8	-0.3	25	In		Exon09-PMO-209	**82.69%**	Exon09-PMO-209	**53.69%**	68.19%	0.21
Fast-Slow	89	Exon27-PMO-001	TTTGTTGGGCCTCTTCTTTAGCTCT	68	44	-7.8	0	25	In		Exon27-PMO-001	**75.90%**	Exon27-PMO-001	**59.51%**	67.70%	0.12
Fast-Slow	90	Exon27-PMO-015	GCTTCTTTTTGTTGGGCCTCTTCTT	69.2	44	-6.6	0	25	In		Exon27-PMO-015	**79.53%**	Exon27-PMO-015	**62.62%**	71.08%	0.12
Fast-Slow	91	Exon27-PMO-031	TCACTTTCGCTTCTTTTTGTTGGGC	71.6	44	-2.8	0	25	In		Exon27-PMO-031	**93.38%**	Exon27-PMO-031	**68.62%**	81.00%	0.18
Fast-Slow	92	Exon27-PMO-055	CAGTAAGGAGTTTCACTTTCGCTTC	65.4	44	-5.2	0	25	In		Exon27-PMO-055	**94.66%**	Exon27-PMO-055	**64.58%**	79.62%	0.21
Fast-Slow	93	Exon27-PMO-287	TTTCCCATTCAGCCTAGTGCAGAGC	72	52	-6.6	-0.5	25	In		Exon27-PMO-287	**69.28%**	Exon27-PMO-287	**51.77%**	60.52%	0.12
Fast-Slow	94	Exon27-PMO-297	TTGCATTTCCCATTCAGCCTAGTGC	72.4	48	-6	-0.2	25	In		Exon27-PMO-297	**14.79%**	Exon27-PMO-297	**8.96%**	11.88%	0.04
Fast-Slow	95	Exon27-PMO-305	AGTCTTGCATTTCCCATTCAGCCTA	69.5	44	-5	0	25	In		Exon27-PMO-305	**0.96%**	Exon27-PMO-305	**0.00%**	0.48%	0.01
Fast-Slow	96	Exon27-PMO-313	CCAAAGTCTTGCATTTCCCATTCAG	70.2	44	-5	0	25	In		Exon27-PMO-313	**30.30%**	Exon27-PMO-313	**18.01%**	24.16%	0.09
