# Supplementary material for: DMD antisense oligonucleotide mediated exon skipping efficiency correlates with flanking intron retention time and target position within the exon
Source: RNA Biol. 2023 Sep 4;20(1):693–702. doi: 10.1080/15476286.2023.2254041 (PMC10481881; doi:10.1080/15476286.2023.2254041)
Supplement: Supplemental Material [file KRNB_A_2254041_SM8312.zip › Supplementary_table2_Primer_sequences.pdf]

Supplementary table S2: primers used (RT-qPCR, RT-PCR and ChIP-qPCR)

Name:	Forward 5'-->3' Sequence:	Reverse 5'-->3' Sequence:	Gene symbol
**RT-qPCR_cDNA-GUSB**	CCGAGTGAAGATCCCCTTTTTA	CTCATTTGGAATTTTGCCGATT	GUSB
**RT-qPCR_cDNA-GAPDH**	CTCTGCTCCTCCTGTTCGAC	ACGACCAAATCCGTTGACTC	GAPDH
**RT-qPCR_cDNA-MYOG**	GCCAGACTATCCCCTTCCTC	GGGGATGCCCTCTCCTCTAA	MYOG
**RT-qPCR_cDNA-MYH3**	CCTGCTGGAGGTGAAGTCTC	GATTGCAGGATCTGGTGGAT	MYH3
**RT-qPCR_cDNA DMD exon 38-39**	attgcttgaaccactggagg	TTTACAGTACCCTCATTGTCTTCAT	DMD
**RT-qPCR_cDNA DMD exon 49-50**	ccagccactcagccagtg	tcagtccaggagctaggtc	DMD
**RT-qPCR_cDNA DMD exon 55-56**	caggatgctacccgtaagga	cgtctttgtaacaggactgc	DMD
**RT-qPCR_cDNA DMD exon 50-52F - 52R**	GACCACTATTGGAGCCTGCAAC	tctagcctcttgattgctgg	DMD

Name:	Forward 5'-->3' Sequence:	Reverse 5'-->3' Sequence:	For exon skip set:	Exp WT size (bp):
RT-PCR DMD H16F2 - H19R	gataactttgcccggtgttg	cattcaccatctgttccacc	Exon 17, 18	407
RT-PCR DMD H19F2 - H25R	actgcaagatgccagcagat	ggctgaattgtctgaatatcactg	Exon 21, 22	978
RT-PCR DMD H47F - H52R	cccataagcccagaagagc	tctagcctcttgattgctgg	Exon 50, 51	798
RT-PCR DMD H54F - ex58R	tagatgtggcaaatgacttgg	gtccttccaaaggctgctct	Exon 55, 57	732
RT-PCR DMD H57F - H61R	ccatttggaagccagttctg	tgagatgctggaccaaagtc	Exon 59	756
RT-PCR DMD H63F2 - H68R	ggaccatcccaaaatgacag	gttacatttggcctgatgctt	Exon 65, 67	694
RT-PCR DMD H69F - H75R	tttctggtcgagttgcaaaa	agaggtgggcatcatttcag	Exon 70	623
RT-PCR DMD H52F - EX58R	ccccagttggaagaactcatt	gtccttccaaaggctgctct	Exon 53	1069
RT-PCR DMD H8F-hEX12R	caaggccacctaaagtgactaaa	gccagtcattcaactctttcag	Exon 09, 10	627
RT-PCR DMD H11F-H15R	GGCCGGGTTGGTAATATTCT	GGCCAGTTTTTGAAGACTTGAT	Exon 14	633
RT-PCR DMD H41F2 - H44R2	gagctgaatgcagtgcgtag	tcagcttctgttagccactga	Exon 42	599
RT-PCR DMD H49F - H53R2	ccagccactcagccagtg	ttgcctccggttctgaagg	Exon 52	540
RT-PCR DMD H25F - H28R	agaatgaagcagagccagag	ctccgccaggaatgttttc	Exon 27	549
RT-PCR DMD H46F - H52R	agaacaaaagaatatcttgtcag	tttgggcagcggtaatgag	Exon 51 in del 48-50	560
RT-PCR DMD H44F - H54R	gcgatttgacagatctgttg	ccaagaggcattgatattctc	Exon 53 in del 45-52	490
